# Age‐dependent aneuploidy in mammalian oocytes instigated at the second meiotic division

**DOI:** 10.1111/acel.13649

**Published:** 2022-06-03

**Authors:** Anna Kouznetsova, Jian Guo Liu, Sonata Valentiniene, Hjalmar Brismar, Christer Höög

**Affiliations:** ^1^ Department of Cell and Molecular Biology Karolinska Institutet Stockholm Sweden; ^2^ Science for Life Laboratory, Department of Applied Physics Royal Institute of Technology Solna Sweden

**Keywords:** age‐dependent aneuploidy, chromosome, meiosis, oocyte, second meiotic division, segregation

## Abstract

Ageing severely affects the chromosome segregation process in human oocytes resulting in aneuploidy, infertility and developmental disorders. A considerable amount of segregation errors in humans are introduced at the second meiotic division. We have here compared the chromosome segregation process in young adult and aged female mice during the second meiotic division. More than half of the oocytes in aged mice displayed chromosome segregation irregularities at anaphase II, resulting in dramatically increased level of aneuploidy in haploid gametes, from 4% in young adult mice to 30% in aged mice. We find that the post‐metaphase II process that efficiently corrects aberrant kinetochore‐microtubule attachments in oocytes in young adult mice is approximately 10‐fold less efficient in aged mice, in particular affecting chromosomes that show small inter‐centromere distances at the metaphase II stage in aged mice. Our results reveal that post‐metaphase II processes have critical impact on age‐dependent aneuploidy in mammalian eggs.

## Graphical abstract

Ageing severely affects the chromosome segregation process in human oocytes resulting in aneuploidy, infertility and developmental disorders. We have studied the second meiotic division in oocytes from aged mice and established that post‐metaphase II processes have critical impact on age‐dependent aneuploidy in mammalian eggs.

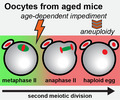

**Abbreviations**
ACA: anti‐centromere antibody
DIC: differential contrast
MTs: microtubules

## 1 INTRODUCTION

Aneuploidy, or an unbalanced number of chromosomes, is a pathological situation for animal cells. Aneuploidy is a common attribute of somatic cancer cells, while aneuploid germ cells contribute to formation of an embryo with an incorrect number of chromosomes, a condition resulting in miscarriage or birth of an individual that show developmental disorders (Potapova & Gorbsky, 2017). Human female germ cells (oocytes) are particularly prone to aneuploidy. Ageing instigates a drastic increase in aneuploidy levels in oocytes from 20–25% at 20 years of age to 50–60% at 40–50 years of age (Gruhn et al., 2019; Nagaoka et al., 2012). Studies have identified multiple examples of chromosomes segregation errors that takes place during the first meiotic division (MI) that could contribute to age‐dependent aneuploidy (Gruhn et al., 2019; Jessberger, 2012; Lister et al., 2010; Nabti et al., 2017; Sakakibara et al., 2015; Yoshida et al., 2015; Zielinska et al., 2015, 2019). Separate from this, 15–40% of aneuploidies in aged human oocytes arise as primary chromosome segregation defects formed during the second meiotic division (MII) after an error‐free MI division (Capalbo et al., 2013; Fragouli et al., 2011; Handyside et al., 2012; Kuliev et al., 2011; Ottolini et al., 2015). We know very little about the nature of the chromosome segregation errors during MII that contribute to age‐dependent aneuploidies in human oocytes (Fragouli & Delhanty, 2013; Webster & Schuh, 2017).

MII is similar to mitosis in the sense that sister chromatids segregate; however, the asymmetric cell division that takes place in MII oocytes gives rise to a large haploid egg and a small deteriorating polar body. The accuracy of the chromosome segregation process depends on correct attachments of spindle microtubules (MTs) to kinetochores, protein complexes formed at the centromeres of sister chromatids (Hinshaw & Harrison, 2018). Kinetochores initially attach to lateral surfaces of spindle MTs (Itoh et al., 2018), a mode of attachment later changed into end‐on kinetochore‐MT attachments when chromosomes congress to the metaphase plate (Itoh et al., 2018). At the metaphase stage, the kinetochores of sister chromatids are attached to opposing proximal spindle poles, forming amphitelic attachments (Hauf & Watanabe, 2004). The sister chromatids are held together by cohesin complex proteins, however, following the degradation of one of the subunits in the cohesin complex at anaphase onset microtubular forces ensure that sister chromatids are pulled towards opposite spindle poles (Morales & Losada, 2018; Oliveira & Nasmyth, 2010; Revenkova et al., 2010). In a situation when a sister kinetochore is connected to both spindle poles by MTs (a geometric configuration called merotelic attachment), it will persist at the spindle midzone until one of the two MTs dissolves, allowing the lagging sister chromatid to segregate to one of the two spindle poles (Gregan et al., 2011; Kouznetsova et al., 2014). Meiotic spindles also contain interpolar MTs that protrude from the spindle pole to the spindle equator, at which they get tethered on overlapping anti‐parallel stretches by a microtubule‐associated protein, Prc1, contributing to spindle stability (Bieling et al., 2010; Duellberg et al., 2013; Tolić, 2018; Yoshida et al., 2020).

We have previously identified an efficient post‐metaphase II correction process that increases the accuracy of the chromosome segregation process in oocytes in young adult mice (Kouznetsova et al., 2019). Here we have analysed the chromosome segregation process during MII in oocytes from aged mice and compared the results to previous data available for young adult mice. We find that oocytes from aged mice show an increased level of abnormal chromosome separation patterns at anaphase II and also a drastically increased level of aneuploidy in haploid gametes. We show that ageing perturbs post‐metaphase correction of aberrant kinetochore‐microtubule attachments, in particular affecting chromosomes that display small inter‐centromere distance at the metaphase II stage.

## 2 RESULTS

### 2.1 A drastic increase in aneuploidy is observed during ageing in MII oocytes

**FIGURE 1:**
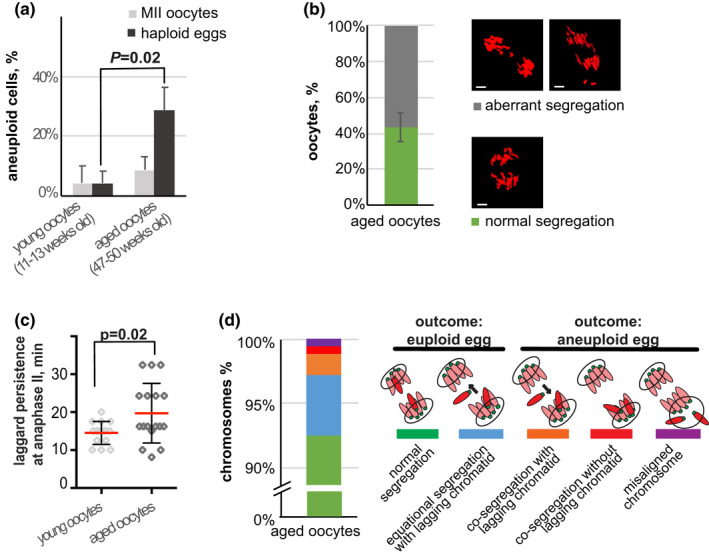
Chromosome segregation errors in oocytes in aged mice increase in an age‐dependent manner. (a) Percentage of aneuploidy (mean ± SD) in wild‐type oocytes in young adult (11–13 weeks old) and aged (47–50 weeks old) mice, from analysis of live oocytes expressing CENP‐C‐EGFP and H2B‐mCherry. An age‐dependent elevation in aneuploidy was observed in haploid eggs (black bars, *n* = 24 for eggs from young adult mice, eight independent experiments and *n* = 57 for eggs from aged mice, 11 independent experiments), but not observed for MII oocytes (grey bars, *n* = 25 for oocytes from young adult mice, five independent experiments and *n* = 35 for oocytes from aged mice, eight independent experiments; *p* = 0.02, chi‐squared test). (b) Percentage of oocytes from aged mice (mean ± SD) that show normal or abnormal segregation patterns at anaphase II, obtained from analysis of live oocytes expressing H2B‐mCherry (*n* = 32, 7 independent experiments). Most oocytes from aged mice showed an abnormal segregation pattern at anaphase II onset. Examples of normal and aberrant segregation patterns are shown to the right. Images are maximum intensity z‐projections of the anaphase stage II in live oocytes, chromosomes (red) are visualized by H2B‐mCherry. Bars, 5 μm. (c) Time (mean ± SD) from the onset of anaphase II until lagging chromatids merge with the chromatin mass, obtained from analysis of live oocytes expressing H2B‐mCherry (*n* = 17 and 15 for oocytes from young adult and aged mice, respectively, from three independent experiments). It took longer time for lagging chromatids to merge with the chromatin mass in oocytes from aged mice comparing to young adult mice (*p* = 0.02, unpaired t test with Welch correction). (d) Percentage of chromosomes in oocytes that display an abnormal segregation pattern at anaphase II in aged mice (*n* = 18 from seven independent experiments). Schematic representations of different types of chromosome segregation patterns are shown to the right, with centromeres labelled in green and chromatin labelled in red. The sister chromatids of analysed chromosome are highlighted, an arrow close to lagging chromatids indicates the direction of segregation at the end of anaphase II. Two segregation patterns do not give rise to aneuploidy, i.e. a normal segregation pattern (green, sister chromatids synchronously separate to opposite spindle poles) and equational segregation of lagging chromatids (blue, a chromatid is initially left behind at the spindle midzone at anaphase II onset, but then correctly segregates to the opposite pole relative to the other sister chromatid). In contrast, in case of sister chromatid co‐segregation with or without lagging chromatid (orange and red, respectively) or anaphase II onset with a misaligned chromosome (violet), these chromosomes give rise to aneuploidy in haploid eggs

We have studied the meiotic chromosome segregation process in 47–50 weeks old female mice (hereinafter referred to as aged mice) in the same strain as was previously used to study the post‐metaphase II correction process in young mice (11–13 weeks old, C57BL/6NCrl). We identified all chromosomes in live cells (where centromeres were labelled by CENP‐C‐EGFP and chromosomes with mCherry‐H2B) and found that aged 47–50 weeks old mice did not display an increased aneuploidy rate in oocytes at completion of MI compared to young adult mice (11–13 weeks old) (Figure 1a, MII oocytes). In contrast, aneuploidy in haploid eggs increased significantly, from 4 ± 4.1% observed at 11–13 weeks, to 29 ± 7.6% at 47–50 weeks (*p* = 0.02, chi‐squared test) (Figure 1a, haploid eggs), on completion of MII. Thus, we find that ageing strongly affects the accuracy of the chromosome segregation process during MII.

### 2.2 Aged female mice exhibit a strongly increased level of chromosome segregation abnormalities at anaphase II

We next analysed the chromosome segregation process during anaphase II by time‐lapse imaging, following chromosomes labelled by mCherry‐H2B. We found that more than half of the oocytes in aged mice (approximately 55%, 21 out of 38 cells) showed severe anaphase II abnormalities, with lagging chromatids found at the spindle midzone or with chromosomes (both sister chromatids) localized to only one of the spindle poles (Figure 1b). We also observed that lagging chromatids in oocytes from aged mice were considerably slower to merge with the chromatin mass at the spindle poles, compared to the situation in oocytes in young adult mice (20 ± 8 min in aged mice and 15 ± 3 min in young adult mice, *p* = 0.02, *t* test with Welch correction), suggesting an age‐dependent defect in segregation of lagging chromatids at anaphase II (Figure 1c).

To find out if the observed chromosome segregation abnormalities at anaphase II in oocytes in aged mice also contributed to aneuploidy, we employed high‐resolution time‐lapse imaging, where centromeres were labelled by CENP‐C‐EGFP and chromosomes with mCherry‐H2B. We determined the segregation pattern of every chromosome in 20 oocytes that showed segregation abnormalities. We found that 7.5% of the chromosomes, on average 1.5 chromosomes in each analysed oocyte, showed signs of segregation irregularities during anaphase II (a total of 30 abnormal chromosomes in 20 aged oocytes, Table S1), including lagging chromatids, chromatid non‐disjunctions with or without visible laggards and anaphase II onset with a misaligned chromosome at the spindle pole (Figure 1d, blue, orange, red and violet bars; see also Video S1–S5 illustrating the behaviour of chromosomes with different segregation patterns). Importantly, about 30% (10 out of 30) of the chromosomes that showed anaphase II abnormalities in aged mice co‐segregated the sister chromatids to one spindle pole, thus giving rise to aneuploidy in haploid gametes (Figure 1d, orange, red and violet bars). We observed no delay in anaphase II onset in oocytes from aged mice that showed aberrant chromosome segregation patterns (Figure S1), suggesting that the spindle assembly checkpoint (Musacchio & Salmon, 2007) was not activated prior to anaphase II onset in response to the segregation errors observed here.

A comparison of the results for aged mice with results previously gathered for young adult mice (Kouznetsova et al., 2019) revealed that whereas 55% of the oocytes in aged mice showed an aberrant segregation pattern at anaphase II, only 15% of the oocytes in young adult mice at anaphase II showed similar aberrant behaviour. Furthermore, 30% of the chromosomes in oocytes from aged mice showed anaphase II abnormalities that contributed to formation of aneuploid gametes, whereas only 10% of the chromosomes in young adult mice contributed to aneuploidy. As a result, the number of chromosomes in oocytes in aged mice that contribute to aneuploidy is increased by an order of magnitude, from 0.1–0.2% in young adult females to 1–2% in aged females. We conclude that ageing contributes to an increased level of chromosome segregation abnormalities at anaphase II in oocytes, as well as an increased number of chromatid co‐segregation events, two events that result in a drastically increased level of aneuploidy in haploid gametes.

### 2.3 Ageing does not affect the metaphase II behaviour of aberrantly segregating chromosomes

**FIGURE 2:**
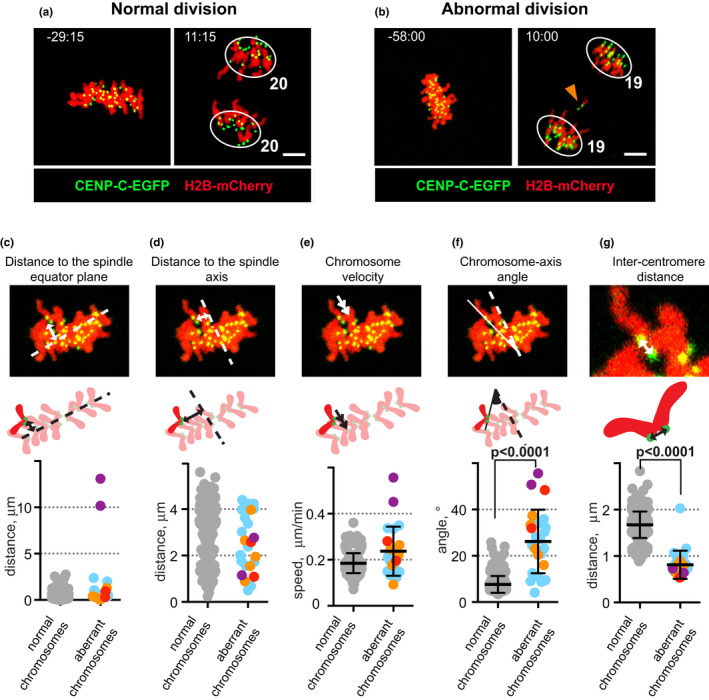
Metaphase II behaviour and the inter‐centromere distance of aberrantly or normally segregating chromosomes in live oocytes from aged mice. (a, b) Time‐lapse imaging of the second meiotic division in representative oocytes from aged mice expressing CENP‐C‐EGFP (centromeres, green) and H2B‐mCherry (chromatin, red) that show a normal segregation pattern (i.e. sister chromatids synchronously become separated to the opposite spindle poles) (a) or an aberrant segregation pattern (b). Images are maximum intensity z projections from representative timepoints at metaphase II and anaphase II. Numbers next to the circles enclosing the segregating chromatids denote the number of chromatids found at the spindle poles. The arrowhead in b labels a lagging chromatid. Bars, 5 μm. See also Videos S1 and S3 for time‐lapse videos of MII division in oocytes shown in a and b. (c–g) Chromosome parameters obtained for each of the 20 chromosomes in oocytes from aged mice that show a normal segregation pattern (*n* = 14, grey circles) and chromosomes that show an aberrant segregation pattern (27 chromosomes from 18 oocytes, the colours correspond to the segregation patterns shown in 1d. The values are averaged for the last 40 min before the anaphase II onset. The illustrative images (maximum intensity z‐projections) and schematic drawings above the charts show chromosomes in red and centromeres in green; spindle equator in c and spindle axis in d and f are indicated by dashed lines; the arrowed lines in the images and schematic drawing indicate chromosome‐equator plane distance in c, chromosome‐axis distance in d, chromosomes velocity in e and inter‐centromere distance in g; the chromosome‐axis angle is highlighted in f. Black lines indicate mean ± SD in the charts for e–g, *p*‐values in f and g are calculated using Mann–Whitney test

What gives rise to the observed abnormal chromosome segregation patterns in oocytes at anaphase II in aged mice? A possibility is that the anaphase II irregularities result from errors introduced at the metaphase II stage. To investigate if this was the case, we visualized chromosomes and centromeres by expressing H2B‐mCherry and CENP‐C‐EGFP proteins in oocytes at MII and performed time‐lapse imaging every 2–3 min at the metaphase II stage. We monitored different aspects of chromosome behaviour, including the positions of chromosomes relative to the spindle equator and the spindle axis, as well as the speed and orientation of chromosomes. We undertook this analysis for all chromosomes in 14 oocytes from aged mice that showed error‐free anaphase II chromosome segregation patterns (Figures 2a, S2A–D and Video S1 for a representative oocyte from aged mice and Figure S2E–H for the average values in all oocytes with a normal segregation pattern; see the quantification details in Experimental Procedures). We also analysed 27 chromosomes that displayed an aberrant segregation pattern at anaphase II in 18 oocytes from aged mice (Figure 2b and Videos S2–S5 for representative oocytes from aged mice, Figure S2I–L for all chromosomes with an aberrant segregation pattern). We observed a similar behaviour at the metaphase II stage for chromosomes that later underwent error‐free or aberrant segregation at the anaphase II stage with the exception of the chromosome‐spindle axis angle, that was elevated from 7.5 ± 2° to 24 ± 12° for chromosomes that would show aberrant segregation at anaphase II (*p* < 0.0001, Mann–Whitney test) (Figure 2c–f). Importantly, the behaviour of the analysed chromosomes in oocytes at the metaphase II stage in aged mice was similar to what previously been documented for chromosomes in oocytes in young adult mice (Table 1).

**TABLE 1:** Meiosis II in mouse oocytes derived from young adult and aged mice

	Young oocytes		Aged oocytes	
	Normal chromosomes	Aberrant chromosomes	Normal chromosomes	Aberrant chromosomes
Max distance from chromosome to the equator plane, μm	1.9 ± 0.6	2.9	2.3 ± 0.5	2.4
Max distance from chromosome to the spindle axis, μm	4.6 ± 0.6	4.5	4.5 ± 0.4	4.4
Chromosome‐axis angle, °	6 ± 2	17 ± 10	7.5 ± 2	24 ± 12
Mean chromosome speed, μm/min	0.2 ± 0.03	0.2 ± 0.04	0.18 ± 0.03	0.22 ± 0.07
Inter‐centromere distance, μm	1.7 ± 0.2	1.3 ± 0.7	1.6 ± 0.3	0.8 ± 0.3

*Note*: Numbers indicate mean ± SD. Data for oocytes from young adult mice is derived from (Kouznetsova et al., 2019).

### 2.4 The inter‐centromere distance for aberrantly segregating chromosomes is reduced in oocytes of aged mice

We next analysed the distance between sister centromeres for chromosomes during metaphase II in oocytes in aged mice using time‐lapse imaging studies. The inter‐centromere distance for chromosomes with a normal or an aberrant segregation pattern was constant between metaphase II and anaphase II onset (Figure S3A,C). Strikingly, chromosomes that showed an aberrant segregation pattern at anaphase II exhibited a 50% reduction in inter‐centromere distance at the metaphase II stage in comparison with chromosomes that segregated normally (reduced from 1.6 ± 0.3 μm to 0.8 ± 0.3 μm, *p* < 0.0001, Mann–Whitney test, Figures 2g and S3C). No difference in inter‐centromere distance for the 9 chromosomes that co‐segregated the sister chromatids and produced aneuploid gametes (violet, red and orange colour in Figures 2g and S3C), compared to the 18 laggard‐producing chromosomes that segregated the sister chromatids equationally in aged mice (blue colour in Figures 2g and S3C), was detected.

The statistical analysis had not revealed any difference in the inter‐centromere distance at the metaphase II stage for chromosomes that segregated normally at anaphase II in oocytes in young adult and aged mice (Table 1). However, the inter‐centromere distance at the metaphase II stage for chromosomes that showed an aberrant segregation pattern at anaphase II was reduced from 1.3 ± 0.7 μm in young adult mice (Kouznetsova et al., 2019) to 0.8 ± 0.3 μm in aged mice (*p* = 0.01, Mann–Whitney test, Table 1).

Interestingly, it has been reported that following inhibition of the kinesin‐like protein Eg5 by monastrol (a treatment that generates monopolar spindles and removes inter‐centromere tension produced by spindle forces), the inter‐centromere distance is increased in mouse MII oocytes in an age‐dependent manner (Merriman et al., 2013; Nabti et al., 2017). We indeed observed a small age‐dependent increase in inter‐centromere distance on a monopolar spindle following treatment of oocytes with monastrol (from 0.7 ± 0.2 at 11–13 weeks old to 0.8 ± 0.3 μm at 47–50 weeks old, *p* = 0.001, Nested ANOVA, Figure S3D). This experiment shows that a functional bipolar spindle is essential to generate a reduced distance between centromeres in oocytes in aged mice.

In summary, we observed a 50% reduced inter‐centromere distance at the metaphase II stage for chromosomes that showed an aberrant segregation pattern at anaphase II compared to chromosomes with a normal segregation pattern at anaphase II in oocytes of aged mice. Furthermore, the inter‐centromere distance at the metaphase II stage was reduced in an age‐dependent manner for chromosomes that showed an aberrant segregation pattern at the anaphase II stage.

### 2.5 Oocytes in aged mice do not display defects in kinetochore‐MT attachments at the metaphase II stage

The reduced inter‐centromere distance observed for aberrantly segregating chromosomes in oocytes from aged mice could result from (i) age‐dependent changes in bi‐directional tension applied to kinetochores by spindle MTs at the metaphase II stage, and/or (ii) anaphase II defects that specifically affect chromosomes with a reduced inter‐kinetochore distance. To evaluate the first alternative, we analysed spindle structure and kinetochore‐MT attachments at the metaphase II stage in fixed young adult and aged oocytes. We could not observe a difference in spindle size in oocytes derived from young adult or aged mice (Figure S4A). Furthermore, no differences in post‐translational modifications of tubulin modulating MT stability, such as α‐tubulin tyrosination and acetylation (Barisic & Maiato, 2016; Gruhn et al., 2019), were observed (Figure S4B,C). We also analysed the integrity of centromeres and kinetochores for evidence of age‐dependent deterioration (Gruhn et al., 2019) using an anti‐centromere antibody (ACA) and an anti‐kinetochore antibody Hec1 but did not observe signs of fragmentation of these structures in oocytes derived from 47–50 weeks old aged mice (Figure S4D,E).

**FIGURE 3:**
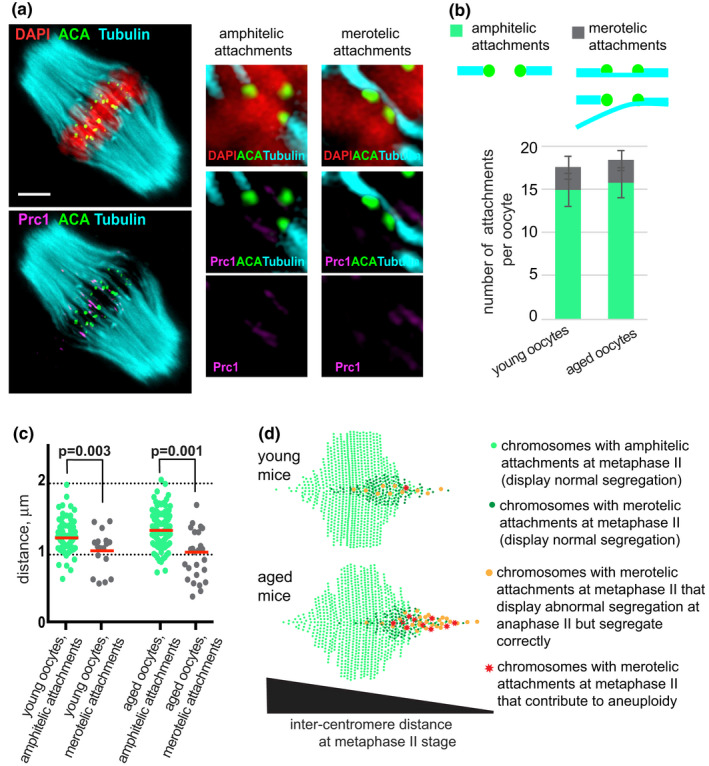
Aged mice preserve kinetochore‐MT attachments at the metaphase II stage, despite multiple segregation abnormalities at anaphase II. (a) Kinetochore‐MT attachments in fixed MII‐arrested oocytes from young and aged mice were visualized using an anti‐tubulin antibody (cyan), an anti‐Prc1 antibody (magenta), an anti‐centromeric ACA antibody (green) and DAPI to label chromatin (red) after removal of non‐kinetochore microtubules by 5 min of cold‐treatment. Image represents maximum intensity projections through all z‐planes containing MTs for a representative oocyte from aged mice. Single z‐planes of representative chromosomes displaying amphitelic and merotelic kinetochore‐MT attachments are shown to the right. Kinetochore‐MT attachments in oocytes from young adult mice look the same as presented images for oocytes derived from aged mice. Scale bar, 10 μm. (b) Number of chromosomes with different types of kinetochore‐MT attachments in oocytes from young (*n* = 7) and aged (*n* = 10) mice. Mean ± SD. The schematic representations of the different types of attachments are shown above the graph (MT, cyan, and kinetochores, green). (c) The inter‐centromere distance for chromosomes with amphitelic (green) and merotelic (grey) attachments in oocytes from young (*n* = 7) and aged (*n* = 10) mice. Red line indicates mean value. *p*‐values are calculated using Mann–Whitney test. (d) Scheme elucidating an age‐dependent inability to resolve aberrant kinetochore‐MT attachments with short inter‐centromere distances. Chromosomes are distributed on a horizontal axis according to their inter‐centromere distances (each chromosome indicated by a circle or a star). Oocytes from aged and young adult mice at the metaphase II stage display a similar distribution of inter‐centromere distances for chromosomes that form amphitelic attachments (light green circles), exhibit the same fraction of chromosomes that form merotelic attachments (dark green, orange circles and red stars) with a similar distribution of inter‐centromere distances. In oocytes from young adult mice, about 1/10 of the chromosomes that form merotelic attachments show an aberrant segregation pattern at anaphase II (orange circles and red star) and approximately 1% of these chromosomes contribute to aneuploidy in haploid eggs (red star). In oocytes from aged mice, almost 1/3 of the chromosomes that form merotelic attachments display an aberrant segregation pattern (orange circles and red stars) and approximately 10% of them contribute to aneuploidy in haploid eggs (red stars). The model is based on extrapolation from the experimental data

We next analysed the nature of the kinetochore‐MT attachments at the metaphase II stage in fixed oocytes using an anti‐Prc1 antibody to identify interpolar MTs (Tolić, 2018), an anti‐α‐tubulin antibody to visualize spindle MTs and ACA to indicate kinetochore positions. We found Prc1 to accumulate in a similar way at the spindle midzone in MII oocytes from young adult and aged mice and the observed patterns of the MTs to be the same (Figure S4F). We removed non‐kinetochore MTs by incubation of oocytes in a cold buffer 5 min before fixation (Figure S4G), followed by image analysis by super‐resolution microscopy, and determined the positions of centromeres and sister centromere pairs for each chromosome in individual oocytes. In most cases, sister kinetochores formed amphitelic attachments to a proximal spindle pole (Figure 3a and green bars in Figure 3b). We observed, however, multiple cases when a single kinetochore engaged in a bilateral association with Prc1‐negative MTs, representing merotelic end‐on attachments (Figure 3a and grey bars in Figure 3b). The number of chromosomes that showed merotelic attachments was similar in oocytes from young adult and aged mice (2.6 ± 1.3 (mean ± SD, *n* = 7) and 2.5 ± 1.2 (mean ± SD, *n* = 10), respectively) (Figure 3b), representing approximately 13% of the chromosomes. The inter‐centromere distance for chromosomes with merotelic attachments was reduced in comparison to chromosomes that showed amphitelic attachments, from 1.4 ± 0.2 μm to 1.0 ± 0.3 μm in oocytes from aged mice (*p* = 0.001, Mann–Whitney test) and from 1.3 ± 0.2 to 1.1 ± 0.3 μm in oocytes in young adult mice (*p* = 0.003, Mann–Whitney test) (Figure 3c).

In summary, we did not observe age‐dependent differences in centromere/kinetochore structures or in the structure and stability of the spindle. Furthermore, the number of merotelic attachments and the inter‐centromere distance for chromosomes in fixed oocytes arrested at the metaphase II stage were the same in oocytes from young adult and aged mice. The inter‐centromere distance at the metaphase II stage for merotelic versus amphitelic attachments in chromosomes was reduced in both young adult and aged mice.

## 3 DISCUSSION

We have here compared the chromosome segregation process at the second meiotic division in oocytes derived from young adult (11–13 weeks old) and aged (47–50 weeks old) C57BL/6NCrl mice. We observed no increase in aneuploidy rate at completion of the first meiotic division for oocytes from aged mice in the studied age range in comparison to young adult females. This result agrees with previously published data that have shown that females on C57Bl6 background are largely resistant to maternal age‐related aneuploidy introduced during MI (Yun et al., 2014). In contrast to this, we find that aneuploidy is increased from 4 ± 4.1% in young adult females to 29 ± 7.6% in aged females at the completion of the second meiotic division. We observed a strongly increased level of chromosomes segregation abnormalities at the anaphase II stage, where aberrant segregation events increased from 15% in young adult mice (Kouznetsova et al., 2019) to 55% in aged mice and the percentage of co‐segregating sister chromatids at anaphase II increased from <10% in young adult mice (Kouznetsova et al., 2019) to 30% in aged mice. In addition, lagging sister chromatids were significantly slower to merge with the chromatin mass at the spindle poles during anaphase II in oocytes in aged mice in comparison to young adult mice.

A synopsis of our findings is shown in Figure 3d. We found that oocytes from young adult and aged mice show the same distribution of inter‐centromere distances and the same percentage of chromosomes (13%) with merotelic attachments at metaphase II (represented by dark green and orange dots and red stars in Figure 3d). 4–5% of all chromosomes in oocytes of aged mice show an abnormal segregation pattern at anaphase II (1–2% in young adult mice (Kouznetsova et al., 2019), orange dots and red stars in Figure 3d), and approximately 1–2% of the chromosomes contribute to aneuploidy in haploid eggs (0.1–0.2% in young adult mice (Kouznetsova et al., 2019), red stars in Figure 3d). Notably, the average inter‐centromere distance at the metaphase II stage was similar for chromosomes with normal segregation pattern in oocytes derived from young adult and aged mice (light and dark green dots in Figure 3d), but chromosomes with an aberrant segregation pattern in live cell imaging studies display a statistically significant reduction in the average inter‐centromere distance (reduced from 1.3 ± 0.6 μm in young adult mice to 0.8 ± 0.3 μm in aged mice; Table 1, orange dots and red stars in Figure 3d). This strongly suggest that the age‐dependent defect identified here primarily affects chromosomes with small inter‐centromere distances.

What gives rise to the increased level of segregation defects at anaphase II and aneuploidy in haploid eggs in aged mice? Kinetochore‐MT attachments or kinetochore/centromere structures at the metaphase II stage do not seem to be affected by ageing, although we cannot completely exclude the possibility that the defects affect only small number of chromosomes and therefore go undetected. However, we observed at the anaphase II stage an increased elevated level of lagging chromatids and co‐segregating sister chromatids in oocytes from aged mice, as well as a temporal delay in completion of anaphase II in oocytes that contained lagging chromatids. We therefore hypothesize that the age‐dependent defect observed here results from a failure to correctly resolve aberrant kinetochore‐MT attachments during anaphase II. Notably, the absence of an age‐dependent increase in aneuploidy after the first meiotic division indicates that anaphase II might be more sensitive to the effect of maternal ageing than anaphase I.

The importance of an anaphase mechanism for correctly segregating lagging chromatids has previously been demonstrated in potoroo Ptk1 somatic cells, fission and budding yeast (Cimini et al., 2004; Courtheoux et al., 2009; Pidoux et al., 2000). Particularly, during spindle poles separation (a stage called anaphase B, which in mouse and human oocytes occurs right after anaphase onset [Fitzharris, 2012]), overlapping interpolar MTs slide apart, pushing chromosomes away from the spindle midzone. During this process, MTs forming amphitelic attachments maintain a constant length (Scholey et al., 2016; Vukušić et al., 2019). Merotelically attached kinetochores on the other hand are connected to both spindle poles and the MTs forming merotelic attachments are forced to elongate. The polymerization rates at the opposite sides can be unequal, and it was modelled that kinetochores move towards the pole with a higher number of MTs attached with a speed proportional to the disbalance in the number of opposing MTs, and only remain at the spindle midzone in case of balanced merotelic attachments, when the ratio of the opposing MTs is equal to 1 (Cimini et al., 2004; Courtheoux et al., 2009). A critical role of the central spindle structure for correction of merotelic attachments has been demonstrated in fission yeast (Courtheoux et al., 2009). The molecular details of how balanced merotelic attachments are resolved in mammalian oocytes remain to be elucidated.

In conclusion, we find that the post‐metaphase II correction process previously described in oocytes in young adult mice remains functional in oocytes of aged mice, however, the efficiency of the process is reduced by an order of magnitude in aged mice. The increased level of aneuploidy in aged haploid gametes is coupled to an impaired ability to resolve aberrant kinetochore‐MT attachments with small inter‐centromere distances. We speculate that the observed age‐dependent defects are due to yet uncharacterized factors located at the spindle midzone that promote segregation of balanced merotelic attachments in oocytes in young adult mice but fail to execute these functions in oocytes in aged mice for chromosomes that show small inter‐centromere distances. Our results should be important to further understand the mechanisms for correcting merotelic attachments in mammalian oocytes and the basis for the chromosome segregation errors at anaphase II that give rise to age‐dependent aneuploidy.

## 4 EXPERIMENTAL PROCEDURES

### 4.1 Mouse oocyte culture and microinjection

The animal experiments were approved by the Stockholm‐North Animal Ethical Committee. Oocytes were taken from 11–13‐weeks old and 47–50‐weeks old wild‐type female mice, produced on a C57BL/6NCrl background. For each experiment, MII ‐arrested oocytes from 2–6 mice were isolated from oviducts after PMS and hCG injections, pooled together and transferred to G‐PGD media (Vitrolife). Ovulation efficiency dropped from 18.4 ± 5.6 oocytes per young female to 3.1 ± 2.7 oocytes per aged female. Mouse oocytes are paused at the metaphase II stage (so called MII arrest) and require artificial activation to progress to the anaphase II stage (Sanders & Jones, 2018). Artificial activation was performed by the addition of 10 mM SrCl_2_ at 37°C. To study the second meiotic division by time‐lapse imaging, a reporter gene coding for histone H2B fused to mCherry was introduced into the experimental mouse strain by backcrossing with reporter mice carrying a H2B‐mCherry fusion gene (Abe et al., 2011). When required, in vitro transcribed 2mEGFP‐CENP‐C mRNA or EGFP‐CENP‐C was microinjected into MII‐arrested oocytes expressing the H2B‐mCherry fusion protein. After 2 h incubation in KSOM medium at 37°C and 5% CO_2,_ oocytes were activated and imaged in G‐PGD media (Vitrolife) supplemented with 10 mM SrCl_2_ at 37°C.

### 4.2 Time‐lapse imaging and stage definition

Analysis of laggard behaviour at anaphase II we performed using a Leica DMI6000 microscope. Red fluorescence and differential contrast (DIC) images were captured every 2.5 min at ×20 magnification for 4 h, for a total of 20–25 1.5‐μm Z‐sections. High‐resolution time‐lapse imaging was performed using the same conditions and simultaneously with the previously reported experiments for young adult oocytes (Kouznetsova et al., 2019). In brief, time‐lapse imaging of oocytes was performed using a Zeiss LSM 780 confocal microscope equipped with a 40× C‐Apochromat 1.2NA water immersion objective (Carl Zeiss) using the 3D multi tracking macro (Rabut & Ellenberg, 2004). We imaged 17–19 consecutive z‐confocal sections (512 × 512 pixels, spaced 1.0 μm), at time intervals of 1.5–3 min. The temporal resolution allowed us to image 5–8 oocytes in the same experiment without apparent phototoxicity effects but also set a limitation for observing movements that lasts for less than 1.5–3 min at metaphase II and anaphase II stages. Anaphase II onset was set to the first time‐frame when the inter‐centromere distance between sister centromeres started to increase.

### 4.3 Oocyte fixation and immunofluorescent imaging

Microtubules were stabilized by fixation in 1.9% formaldehyde in BRD80 buffer (80 mM K‐PIPES, 1 mM MgCl_2_, 1 mM EGTA, pH 6.8), as described previously (Kouznetsova et al., 2019). Where stated, a 5 min long cold treatment in 80 mM K‐PIPES, 1 mM MgCl_2_, pH 7.4 was applied. For the studies of centromere and kinetochore structure, oocytes from aged and young mice were fixed in 1% PFA supplemented with 0.15% Triton X‐100 as described in (Kouznetsova et al., 2014). Metaphase II oocytes were fixed 1–2 h after isolation from the oviducts. Oocytes derived from aged and young adult mice were placed on the same slide to reduce condition variability. The antibodies used were ACA (Antibodes Inc.) at a 1:100 dilution, tubulin‐FITC (Sigma) 1:2000, mouse monoclonal anti‐acetylated α ‐tubulin (Lys‐40) (Sigma) 1:200; rat monoclonal anti‐tyronizinated α‐tubulin (Sigma) 1:200, mouse monoclonal Prc1 (Santa‐Cruz) 1:100 and rabbit polyclonal Hec1 (Abcam) 1:50. The secondary antibodies were goat‐anti‐rabbit Cyanine3 (Invitrogen) at 1:500 dilution, goat‐anti‐mouse Cyanine3 (Invitrogen) 1:500, goat‐anti‐rat Cyanine3 (Invitrogen) 1:500 and donkey‐anti‐human Alexa 647 (Invitrogen) 1:1000. Oocytes were mounted in ProLong Diamond (Thermo Fisher Scientific). Images of kinetochore‐MT attachments, centromeres and kinetochores were collected using a Zeiss 900 with an Airyscan module at 63×/1.4 NA objective, processed by ZEN blue software with Airyscan processing module and Imaris 9.4 software (Bitplane).

### 4.4 Centromere tracking and image quantification

Centromere tracking was performed with Imaris 5.7 image analysis software (Bitplane) using a tracking procedure as described previously (Kitajima et al., 2011; Kouznetsova et al., 2019). In brief, the cubic splines with 0.35 smoothing parameter were fitted to centromere tracks, and smoothed values were used for the calculations. To find the position of the centre, we calculated the centroid of all centromeres. The spindle axis was defined as a line that had an averaged orientation including all aligned chromosomes. The spindle axis went through the centre, the spindle equator plane was perpendicular to the spindle axis and crossed it at the centre. Chromosome position was defined as a midpoint of the line connecting two sister centromeres. Chromosome orientation was defined as the angle between the spindle axis and the line that connected sister centromeres. The oocytes were averaged for the last 40 min before anaphase II onset. Data processing and plotting were performed using Fiji (Schindelin et al., 2012), MATLAB (Bitplane) and GraphPad Prism (GraphPad Software, Inc.). Spindle length was measured between the spindle poles and spindle width was measured at 1 μm distance from the spindle pole towards spindle equator plane on the spindles visualized by anti‐tubulin antibody. The measurements were performed using a Fiji software in fixed oocytes with spindle axis positioned parallel to the imaging plane. The inter‐centromere distances for chromosomes with merotelic attachments and chromosomes with aberrant segregation patterns shown in Figure 3d were obtained by extrapolation of the experimental distributions of inter‐centromere distances reported in Figures 2g and 3c; the data for the inter‐centromere distances in oocytes from young mice was taken from (Kouznetsova et al., 2019). To facilitate the comparisons between oocytes in young and aged mice, the initial number of all chromosomes was equalized to 1000 in both cases.

### 4.5 Statistical analysis

Data are presented as means or median values, as indicated, from at least three independent experiments. We used *t* test for comparisons between the two groups with applied Welch correction if the standard deviation differed significally between the compared groups, Mann–Whitney test for comparing data that had not passed D'Agostino & Pearson omnibus normality test, chi‐squared test to compare frequencies, and nested ANOVA to compare the chromosome parameters between oocytes. The statistical analysis was performed by GraphPad Prism and R software (https://www.r‐project.org), and a *p* value of less than 0.05 was considered statistically significant.

## AUTHOR CONTRIBUTIONS

AK designed and conducted the experiments, analyzed the data and wrote the manuscript; HB provided support with the live‐imaging experiments, JGL and SV helped with experimental work; CH designed experiments and wrote the manuscript.

## CONFLICT OF INTERESTS

The authors declare no competing interests.

## Supporting information


Video S1

Video S2

Video S3

Video S4

Video S5

Supplementary Material

## Data Availability

The data that support the findings of this study are available from the corresponding author upon reasonable request.
