# Supplementary material for: Age‐dependent aneuploidy in mammalian oocytes instigated at the second meiotic division
Source: Aging Cell. 2022 Jun 3;21(7):e13649. doi: 10.1111/acel.13649 (PMC9282850; doi:10.1111/acel.13649)
Supplement: Supplementary file 6 — Supplementary Material [file ACEL-21-e13649-s004.pdf]

Supplementary Figure 1

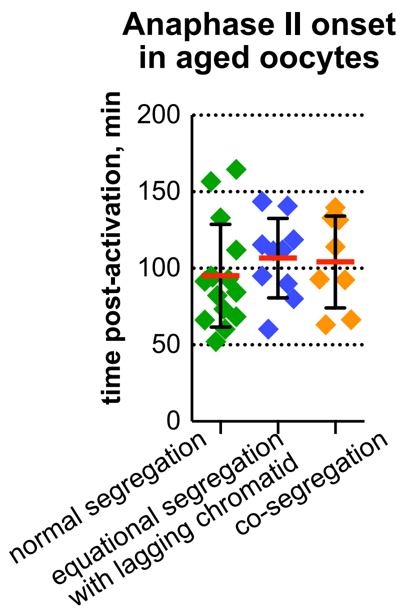

Figure S1. Onset of anaphase II in oocytes from aged mice.

The timing of anaphase II onset is not delayed in oocytes with lagging chromatids that undergo equational segregation (blue, n=10) or with co-segregating chromosomes (orange, n=8) in comparison to oocytes that show a normal segregation pattern (green, n=15). Results from 8 independent experiments. Red line indicates mean values, error bars indicate SDs.

Supplementary Figure 2

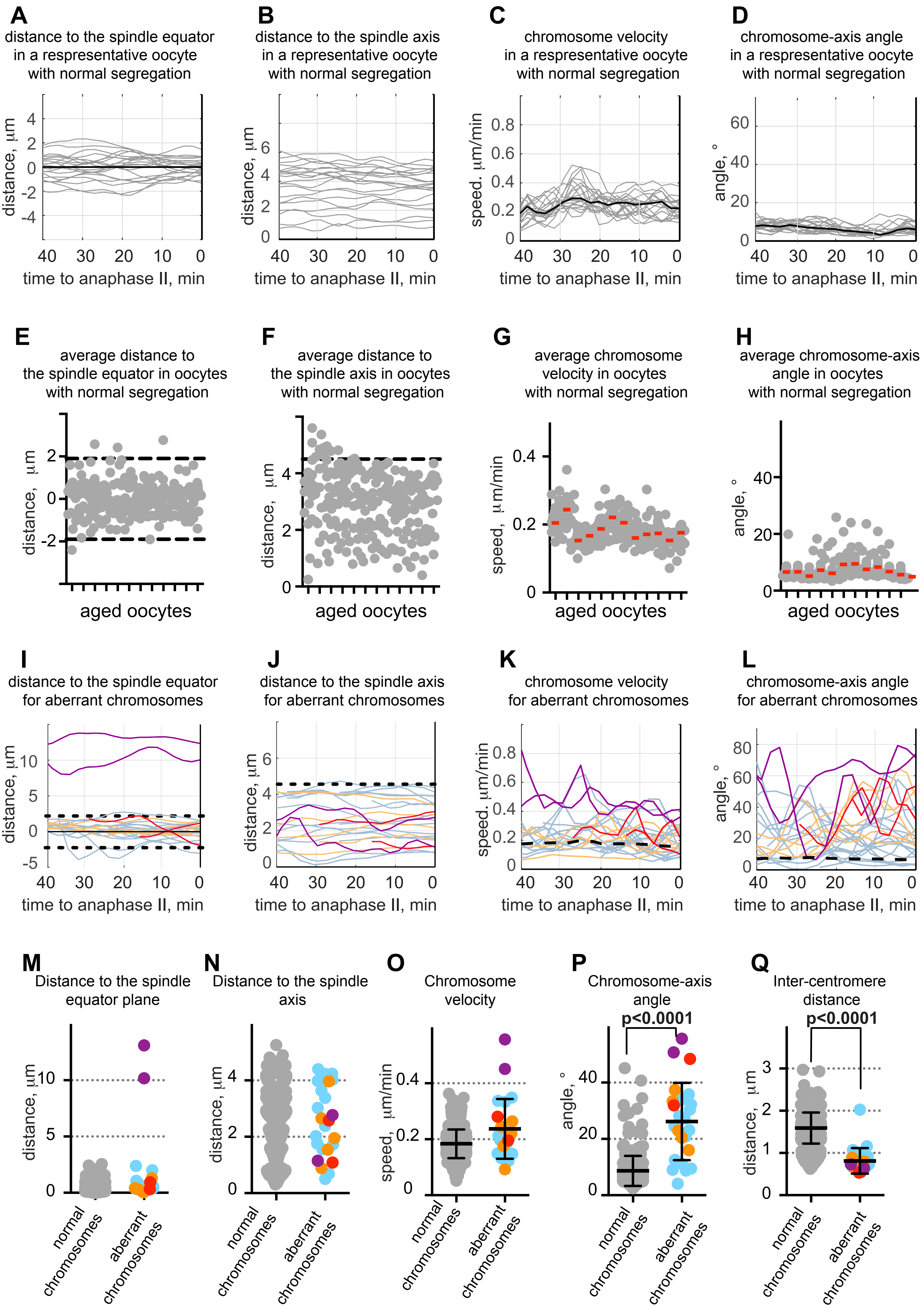

Figure S2. Chromosome behaviour from metaphase II to anaphase II onset in aged oocytes with normal and aberrant segregation patterns

(A-D) Chromosome parameters obtained for a representative oocyte from aged mice with a normal segregation pattern (also shown in **Fig.2A** and **Video S1**) are displayed in **A-D** on the vertical axis and time in min is shown on the horizontal axis relative to the anaphase II onset. Thin grey lines in **A-D** represent individual chromosomes; thick black line in **C-D** shows the mean value for each timepoint.

(**E-H)** Mean parameters calculated for 40 min before anaphase II onset for each chromosome in oocytes that show a normal segregation pattern, representing the variability between chromosomes. Individual oocytes are arranged along the horizontal axis (n=14, 8 independent experiments), grey circles represent individual chromosomes (n=20 in each oocyte). Dashed lines in **E** and **F** represent the mean thickness and radius, respectively, of the metaphase II plate. Red lines in **G** and **H** indicate the median values for each oocyte.

(I-L) Chromosome parameters (as indicated above the charts) obtained for aberrantly segregating chromosomes are shown on the vertical axis, time in min on the horizontal axis relative to anaphase II onset. The colours correspond to the segregation patterns shown in **Fig.1C**. Black dotted line in **I-J** indicates the average size of the metaphase plate, the dashed line in **K-L** indicates mean values for normally segregating chromosomes.

(M-Q) Chromosome parameters obtained for every chromosome in oocytes that show an aberrant segregation pattern (n=18). Normally segregating chromosomes are indicated by grey circles and chromosomes that show an aberrant segregation pattern (27 chromosomes from 18 oocytes) are indicated by the colours corresponding to the segregation patterns shown in **Figure1D**. Please note that parameters of normally segregating chromosomes in oocytes where we observe a few aberrantly segregating chromosomes (shown by grey circles in **M-Q**) and in oocytes where all chromosomes segregate normally (grey circles in Fig. 2 C-G) are almost identical. The values are averaged for the last 40 min before the anaphase II onset. Black lines indicate mean±SD in the charts for **O-Q,** p-values in **P** and **Q** are calculated using Mann-Whitney test.

Supplementary Figure 3

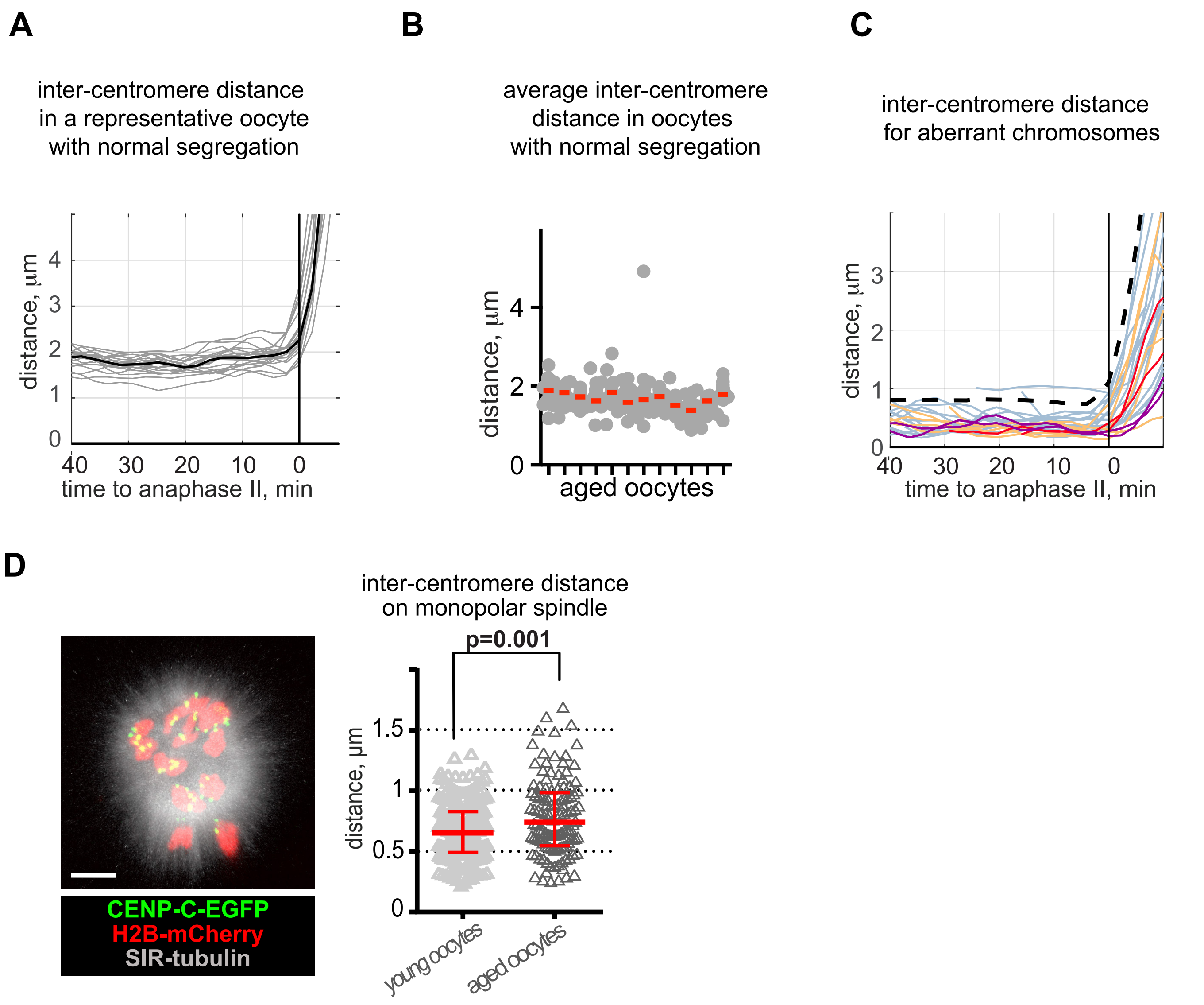

Figure S3. The inter-centromere distances for chromosomes in live oocytes from aged mice

(A) Changes in inter-centromere distance for all 20 individual chromosomes (thin grey lines) in a representative oocyte with a normal segregation pattern (shown in Fig.2A and Video S1). Horizontal axis shows time to anaphase II onset in min.

(B) Mean inter-centromere distance (on the vertical axis), measured for 40 min before anaphase II onset, is relatively constant for all oocytes. Red lines indicate the median values for each oocyte.

(C) Changes in the inter-centromere distances for 27 aberrant chromosomes. The colours correspond to the segregation patterns shown in Fig.1C. Dashed line indicates the mean inter-centromere distance for normally segregating chromosomes. Horizontal axis shows time to anaphase II onset in min.

(D) A maximum z-projection image of chromosome configuration in a representative live oocyte after monastrol addition and formation of a monopolar spindle (left). The chromosomes are labelled by H2B-mCherry (red), the spindle is visualized by SIR-tubulin (white) and the centromeres are labelled by CENP-C-EGFP (green). The image represents maximum intensity projection through all z-planes containing microtubules. Bar, 5 µm. (right) The inter-centromere distance on a monopolar spindle for all chromosomes in oocytes from aged mice (dark grey triangles, n=6) is slightly elevated in comparison with inter-centromere distance found in young oocytes (light grey triangles, n=11, p=0.001, Nested ANOVA).- Data from 3 independent experiments. Red lines indicate mean ±SD.

Supplementary Figure 4

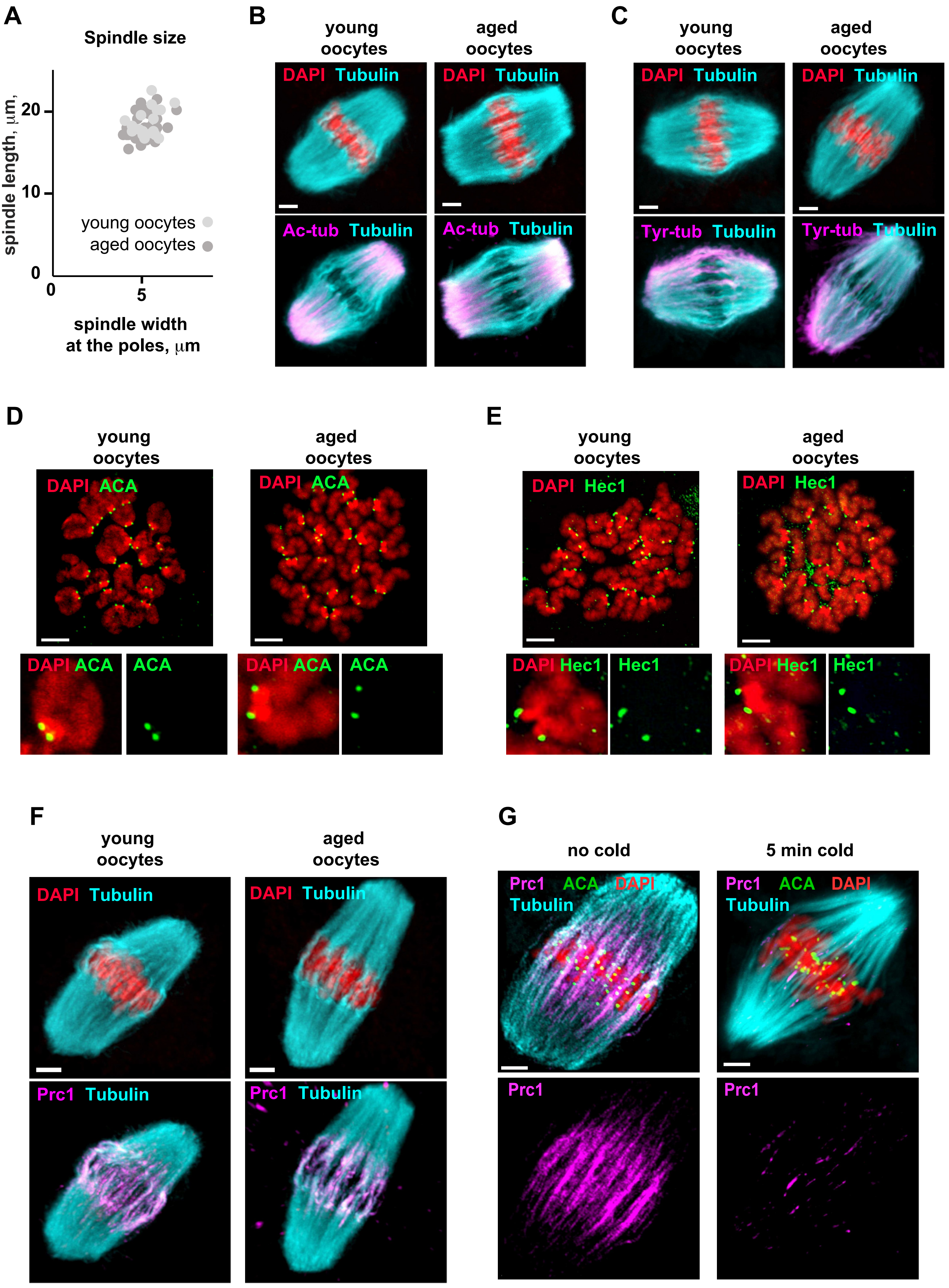

Figure S4. Spindle size, tubulin post-translational modifications, centromere/kinetochore structure and Prc1 localization at the metaphase II stage in oocytes from young and aged mice

(**A**) Spindle pole-to-pole length (shown on vertical axis) and spindle width (on horizontal axis) in oocytes from young (light grey, n=20) and aged (dark grey, n=31) mice, 3 independent experiments.

(B-C) MT acetylation (**B**) and tyrosination (**C**) in MII oocytes from young and aged mice. Oocytes from young (on the left) and aged (on the right) mice were fixed and stained with antibodies recognizing acetylated (Ac-tubulin, magenta in **B**) and tyrosinated forms of α-tubulin (Tyr-tubulin, magenta in **C**). Unmodified α-tubulin was labelled by anti-tubulin antibody (cyan), and chromosomes were stained by DAPI (red). Images are z-projections through all tubulin-containing z-planes. Bars, 5μm.

(D-E) Centromere and kinetochore structures in MII oocytes from young adult and aged mice. Oocytes from young adult (on the left) and aged (on the right) mice were stained with an ACA antibody (ACA, green in **D**) to label the centromere structure, an anti-Hec1 antibody to label the kinetochore structure (Hec1, green in **E**) and DAPI (red) to label chromatin. Enlarged images of representative chromosomes and their centromeres are shown below the oocytes. Images are single z-planes containing centromeres and kinetochores. Bars, 5μm.

(F) Prc1 in MII oocytes from young and aged mice. Oocytes from young adult (on the left) and aged (on the right) mice were fixed and stained with an anti-Prc1 antibody (magenta). Tubulin was labelled by an anti-tubulin antibody (cyan), and chromosomes were stained by DAPI (red). Images are z-projections through all tubulin-containing z-planes. Bars, 5μm.

(G) Prc1 staining at the spindle midzone disappears following cold treatment. Oocytes from young mice were incubated in a cold buffer for 5 min (right column) before fixation; left column shows untreated oocytes. Fixed oocytes were stained with an anti-Prc1 antibody (magenta), an anti-tubulin antibody (cyan) and an anti-centromere antiserum (ACA, green). Chromosomes were labelled with DAPI (red). Bars, 5μm.

Video S1.

Time-lapse video of MII in oocytes in aged mice with a normal segregation pattern. Chromosomes (red) were labelled by H2B-mCherry, centromeres (green) were labelled by CENP-C-EGFP. Sister centromeres of one of the normally segregating chromosomes are indicated by blue spheres. Time: mm:sec relative to anaphase II onset. Bar: 10μm.

Video S2.

Time-lapse video of MII in oocytes in aged mice with an aberrant segregation pattern, where sister chromatids for one chromosome undergo an equational segregation with a chromatid lagging at the midzone at anaphase II. Chromosomes (red) were labelled by H2B-mCherry, centromeres (green) were labelled by CENP-C-EGFP. Sister centromeres of the aberrantly segregating chromosome are indicated by blue spheres. Time: mm:sec relative to anaphase II onset. Bar: 10μm.

Video S3.

Time-lapse video of MII in oocytes in aged mice with an aberrant segregation pattern, where both sister chromatids co-segregate to the same spindle pole (with a chromatid lagging at the midzone at anaphase II) and produce an aneuploid egg. Chromosomes (red) were labelled by H2B-mCherry, centromeres (green) were labelled by CENP-C-EGFP. Sister centromeres of the aberrantly segregating chromosome are indicated by blue spheres. Time: mm:sec relative to anaphase II onset. Bar: 10μm.

Video S4.

Time-lapse video of MII in oocytes in aged mice with an aberrant segregation pattern, where both sister chromatids for one chromosome co-segregate to the same spindle pole (without a chromatid lagging at the midzone at anaphase II onset) and produce an aneuploid egg. Chromosomes (red) were labelled by H2B-mCherry, centromeres (green) were labelled by CENP-C-EGFP. Sister centromeres of the aberrantly segregating chromosome are indicated by blue spheres. Time: mm:sec relative to anaphase II onset. Bar: 10μm.

Video S5.

Time-lapse video of MII in oocytes in aged mice with an aberrant segregation pattern, where chromosome segregation at anaphase II starts with one of the chromosomes positioned close to the spindle pole. Chromosomes (red) were labelled by H2B-mCherry, centromeres (green) were labelled by CENP-C-EGFP. Sister centromeres of the misaligned chromosome are indicated by blue spheres. Time: mm:sec relative to anaphase II onset. Bar: 10μm.

Supplementary Table 1. Synthesis of data for each individual oocyte with the aberrant segregation pattern

	Experiment	Oocyte	Anaphase II onset time, min post-activation	Amount of chromosomes with abnormal segregation	Defect for each chromosome with the aberrant segregation	Gain or loss of chromatid in the egg	Distance to the spindle equator, μm	Distance to the spindle axis, μm	Chromosome velocity, μm/min	Chromosome-axis angle, °	Inter-centromere distance, μm
1	EXP1	EXP1-01	66,5	2	Chromatid co-segregation with lagging chromatid	loss	0,41	1,54	0,26	37,35	0,60
2					Chromatid co-segregation without lagging chromatid	gain	0,93	2,58	0,28	31,91	0,55
3	EXP2	EXP2-02	111,3	1	Equational segregation with lagging chromatid		0,28	3,98	0,21	20,85	0,67
4		EXP2-03	202,3	2	Equational segregation with lagging chromatid		n/d*	n/d	n/d	n/d	n/d
5					Chromatids co-segregation with lagging chromatid	gain	n/d	n/d	n/d	n/d	n/d
6		EXP2-04	139,7	1	Chromatids co-segregation with lagging chromatid	loss	1,31	2,66	0,22	20,42	0,79
7	EXP3	EXP3-05	112	1	Equational segregation with lagging chromatid		0,16	0,50	0,24	9,52	0,74
8		EXP3-06	118,8	1	Equational segregation with lagging chromatid		0,10	3,02	0,20	8,77	0,99
9		EXP3-07	95	1	Equational segregation with lagging chromatid		0,34	4,40	0,21	24,52	0,71
10	EXP4	EXP4-08	92,8	1	Anaphase onet with misaligned chromosome	gain	10,17	5,15	0,56	50,72	0,64
11		EXP4-09	131,6	4	Equational segregation with lagging chromatid		0,42	2,02	0,19	32,24	0,78
12					Equational segregation with lagging chromatid		0,61	0,69	0,24	23,12	0,83
13					Equational segregation with lagging chromatid		2,01	1,76	0,15	11,13	1,16
14					Anaphase onet with misaligned chromosome	loss	13,10	6,77	0,45	55,61	0,73
15		EXP4-10	63,1	2	Equational segregation with lagging chromatid		0,37	3,38	0,18	27,28	0,73
16					Chromatids co-segregation without lagging chromatid	gain	0,32	1,09	0,20	48,38	0,54
17	EXP5	EXP5-11	143,5	1	Equational segregation with lagging chromatid		n/d	n/d	n/d	n/d	n/d
18		EXP5-12	115,4	1	Equational segregation with lagging chromatid		0,88	1,74	0,16	35,84	0,62
19		EXP5-13	133,0	2	Equational segregation with lagging chromatid		0,87	2,48	0,21	9,93	1,04
20					Chromatids co-segregation with lagging chromatid	loss	0,29	1,95	0,18	16,00	0,79
21		EXP5-14	80,2	1	Equational segregation with lagging chromatid		0,41	3,75	0,19	0,28	1,08
22	EXP6	EXP6-15	140,7	1	Equational segregation with lagging chromatid		1,06	4,24	0,33	9,07	0,92
23		EXP6-16	90	2	Equational segregation with lagging chromatid		2,39	3,64	0,35	34,06	0,59
24					Equational segregation with lagging chromatid		0,36	4,23	0,34	12,56	0,66
25		EXP6-17	114	2	Equational segregation with lagging chromatid		0,27	3,72	0,19	6,15	1,11
26					Chromatids co-segregation with lagging chromatid	gain	0,30	3,97	0,25	33,41	0,64
27	EXP7	EXP7-18	60,2	1	Equational segregation with lagging chromatid		0,54	4,03	0,15	4,11	2,03
28		EXP7-19	60,2	2	Equational segregation with lagging chromatid		0,26	2,39	0,13	28,17	1,07
29					Equational segregation with lagging chromatid		0,76	1,56	0,12	30,41	0,84
30		EXP7-20	92,5	1	Chromatids co-segregation with lagging chromatid	gain	0,05	0,89	0,09	23,03	0,89

* n/d - not determined
